# One month in after six months out: the need for medical students to stay resolute

**DOI:** 10.1080/10872981.2020.1830682

**Published:** 2020-10-05

**Authors:** Ben Walter

**Affiliations:** Faculty of Life Sciences and Medicine, King’s College London School of Medical Education, London, UK

The COVID-19 crisis has disrupted the education of medical students significantly. Medical schools have shifted the methods of teaching to online and assessment to open book [1] – adapting quickly to a more virtualised world. Whilst no one is to blame for such circumstances, it has left a hole to fill. Whilst the progress in virtual teaching has been encouraging, learning to become a doctor has classically been in a clinical environment, physically examining patients and undertaking procedures [2]. Therefore, for students that have missed months of clinical placement and a chance to be assessed on their clinical skills (with this summer’s OSCEs being cancelled), it would be understandable that an underlying feeling of anxiety accompanied their return to hospital.

Speaking on a personal level, one of the biggest challenges upon my return has been the knowledge base that has been lost – six months of not studying medicine has undoubtedly caused some knowledge gaps that require frequent reminders. Unfortunately, this can lead to a loss of enthusiasm from teachers who can become noticeably less engaged in our teaching.

Additionally, managing the expectations from senior doctors has proved difficult. Doctors will remember roughly how much they knew by the time they reached the penultimate year of medical school – however, the curriculum has changed significantly since their studies and they were also not disrupted by a global pandemic. This dynamic of explaining why we have not learnt a certain topic or clinical skill can be tough. Whilst many doctors are kind and understanding, to some it can sound like an excuse and shying away from our responsibilities. Consequently, there is a possibility for medical students to be made to feel ashamed for their lack of knowledge or skill. Shame is a well-established issue in medical students’ training and studies have shown it can lead to students displaying shame-avoiding behaviours [3], which can manifest as reduced clinical attendance and stagnation of learning.

Recent literature has also shown the importance of the ‘can be’ mindset rather than what we ‘are’ right now [4]. For medical students, skills such as formulating differential diagnoses and management plans can be a real test and often leads to failure (or incompleteness). Therefore, to prevent discouragement, the emphasis must be on the process of improvement, rather than the short-term outcomes. However, this developmental mindset cannot thrive if teachers do not employ that same outlook. Therefore, it is of utmost importance that medical educators are constantly developing themselves and ensuring a space is created which allows medical students to thrive. This, in turn, must be met by hard work and dedication from said students – the combination of these two factors will ensure the physicians of the future are of just as high a quality, in spite of the disruptions caused by the COVID-19 pandemic.
